# Supplementary material for: Targeting YAP to overcome acquired resistance to ALK inhibitors in ALK‐rearranged lung cancer
Source: EMBO Mol Med. 2019 Oct 21;11(12):e10581. doi: 10.15252/emmm.201910581 (PMC6895608; doi:10.15252/emmm.201910581)
Supplement: Supplementary file 1 — Appendix [file EMMM-11-e10581-s001.pdf]

Targeting YAP to overcome acquired resistance to ALK inhibitors in *ALK*-rearranged lung cancer

Mi Ran Yun [1,2*], Hun Mi Choi [2*], You Won Lee [2], Hyeong Seok Joo [1], Chae Won Park [2], Jae Woo Choi [3,4], Dong Hwi Kim [1], Han Na Kang [1,2], Kyoung-Ho Pyo [2], Eun Joo Shin [2], Hyo Sup Shim [5], Ross A Soo [6], James Chih-Hsin Yang [7], Sung Sook Lee [8], Hyun Chang [9], Min Hwan Kim [2], Min Hee Hong [2], Hye Ryun Kim [2], Byoung Chul Cho [2§]

Appendix - Table of contents

・Appendix Figure S1.

・Appendix Figure S2:

・Appendix Figure S3:

・Appendix Figure S4:

・Appendix Figure S5:

・Appendix Figure S6:

・Appendix Figure S7:

・Appendix Figure S8:

・Appendix Figure S9:

・Appendix Figure S10:

・Appendix Figure S11:

・Appendix Table S1:

・Appendix Table S2:

・Appendix Table S3:

・Appendix Table S4:

・Appendix Table S5:

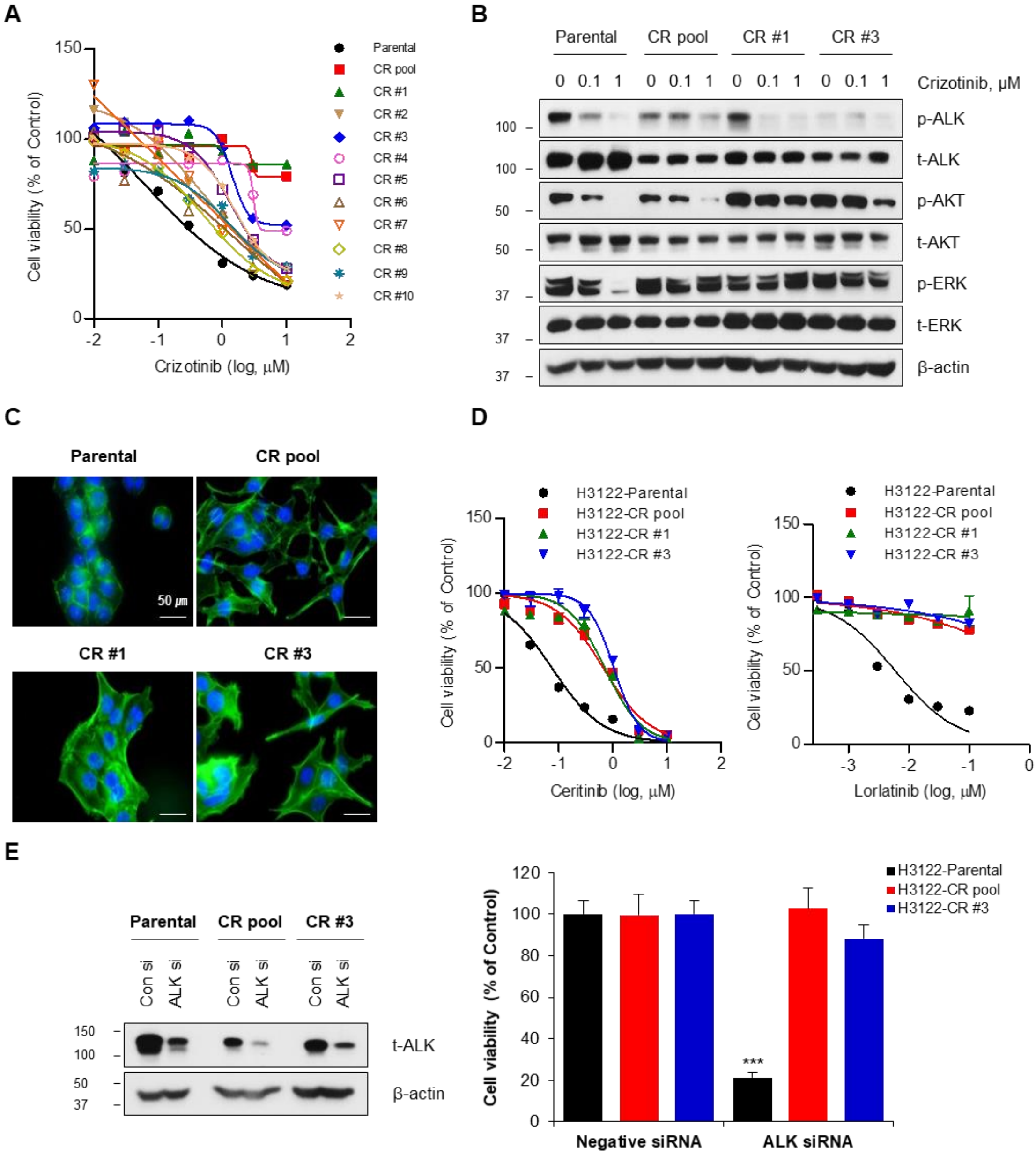

Appendix Figure S1. Independence of ALK signaling in Crizotinib-resistant H3122 cells.

A Cell viability curve in response to increasing doses of crizotinib in H3122 cells (parental) and crizotinib resistant cells using MTT assay.

B Representative immunoblots for the indicated proteins in lysates of cells treated with crizotinib for 6 h. *n* = 3

C Immunofluorescent staining of phalloidin (green) and DAPI (blue) in cells. *n* = 3 Scale bars, 50 μm

D Cell viability curve in response to increasing doses of ceritinib or lorlatinib in parental and resistant cells using MTT assay.

E Left, ALK silencing was confirmed by immunoblot analysis in parental and CR cells transfected with either negative or ALK siRNA (50 nM). Right, The bar chart showing cell viability in ALK siRNA- transfected parental and CR cells. ***$P < 0.001$ compared with negative siRNA.

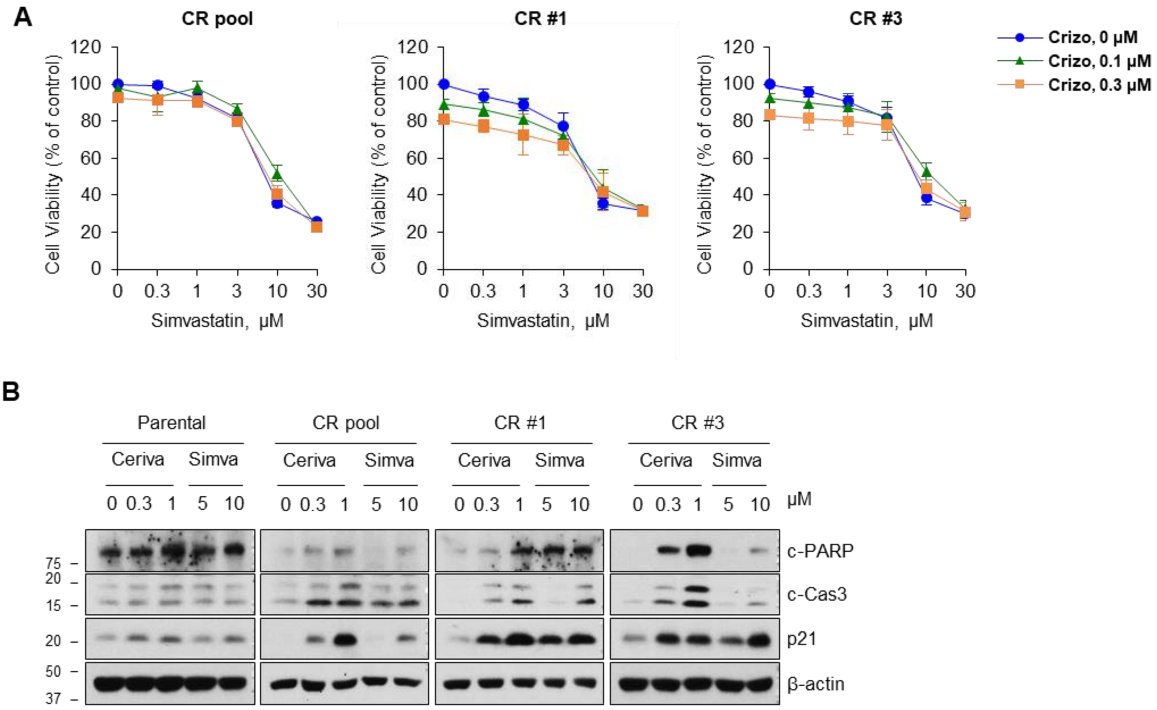

Appendix Figure S2. ***In vitro*** **anti-proliferative effect of simvastatin.**

A Cell viability curve in response to combined treatment of simvastatin and crizotinib in parental and CR cells using MTT assay

B Representative immunoblots for the indicated proteins in lysates of cells treated with either cerivastatin (Ceriva) or simvastatin (Simva) for 24 hours (n = 3). Blots are representative of three independent experiments.

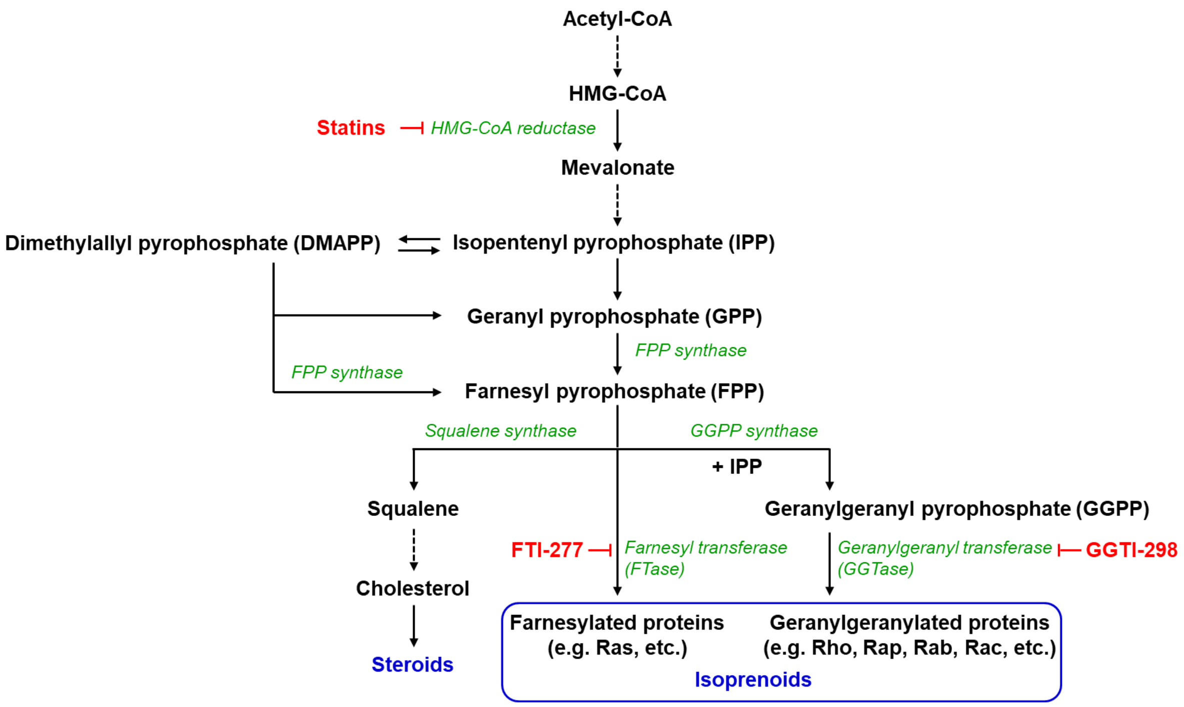

Appendix Figure S3. Schematic diagram of the mevalonate (MVA) pathway.

The MVA pathway produces sterols and isoprenoids, which derived from the farnesyl and geranylgeranyl groups, respectively. Schematic diagram is modified by reference to Mullen *et al* (2016) and Iannelli *et al* (2018). Dashed arrows represent multiple steps omitted for simplicity in the MVA pathway. Final metabolites of MVA pathway are shown in blue, and metabolic enzymes are shown in green.

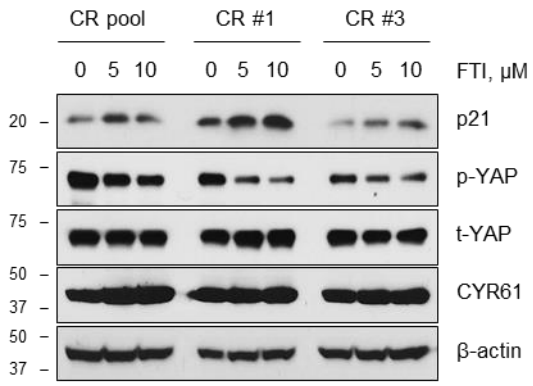

Appendix Figure S4. Effect of FTI-277 in CR cells.

Representative immunoblots for the indicated proteins in lysates of cells treated with FTI-277 for 24 h. Blots are representative of three independent experiments.

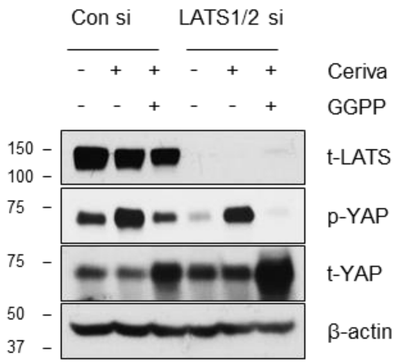

Appendix Figure S5. Involvement of LATS1/2 on GGPP-mediated YAP activation in CR pool cells.

Representative immunoblots for the indicated proteins after treatment of cerivastatin (1 μM) alone or with GGPP (10 μM) for 24 h in CR pool cells transfected with siRNAs targeting negative control (Con) and LATS1/2.

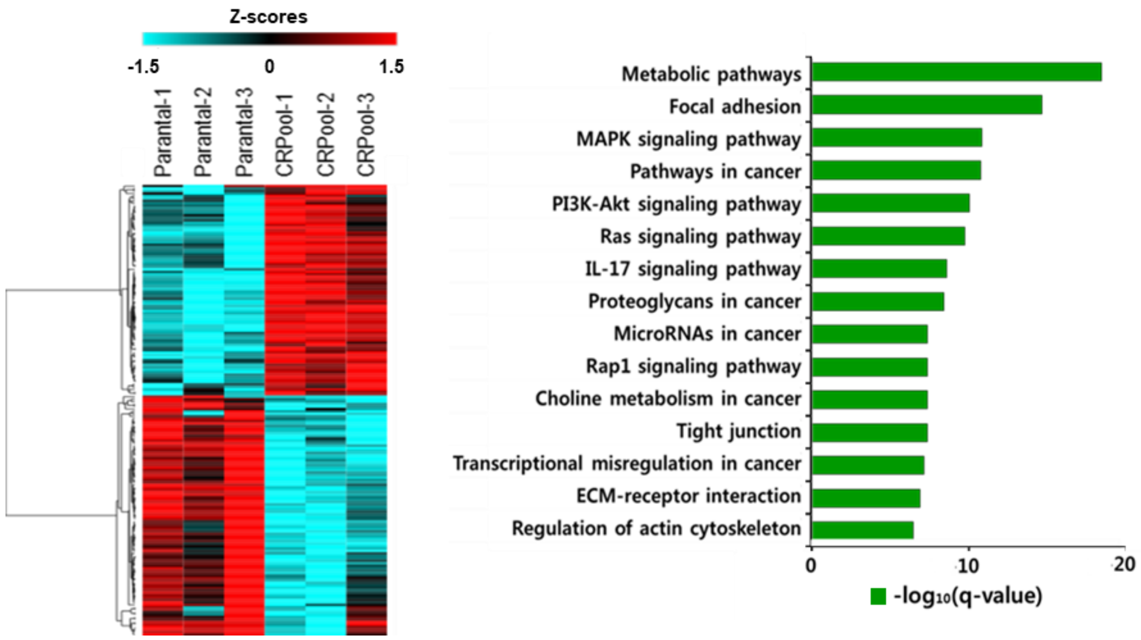

Appendix Figure S6. The differential gene expression in ALK-TKI resistant cells.

Left, Heat-map showing statistically significant DEGs between parental and CR pool. Right, the bar plots showing the top 15 signaling pathways of DGE by KEGG pathway enrichment analysis.

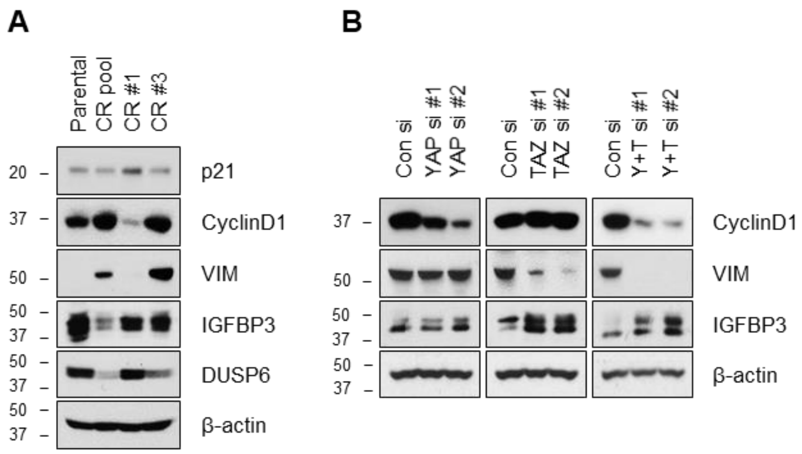

Appendix Figure S7. Effect of inhibition of YAP and TAZ on expression of YAP associated genes in CR pool cells.

A Representative immunoblots for the indicated proteins in basal lysates of CR cells compared to parental cells.

B Representative immunoblots for the indicated proteins in lysates of CR pool cells transiently transfected with either negative control siRNA (Con si), TAZ siRNAs (two sets of siRNAs against TAZ; TAZ si#1 and TAZ si#2), or combination of TAZ siRNAs with YAP si #2 (Y+T si #1 and Y+T si #2).

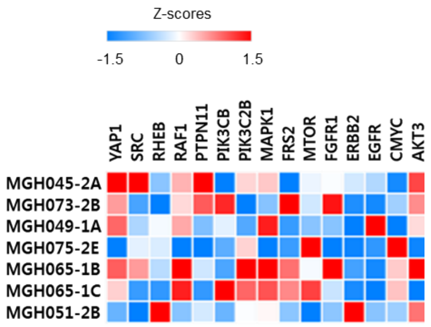

Appendix Figure S8. Involvement of YAP in ALK-TKI resistant cells.

Heat-map showing the average z-score of top candidate genes from previously published the pooled shRNA screen data in ALK-inhibitor-resistant PDCs.

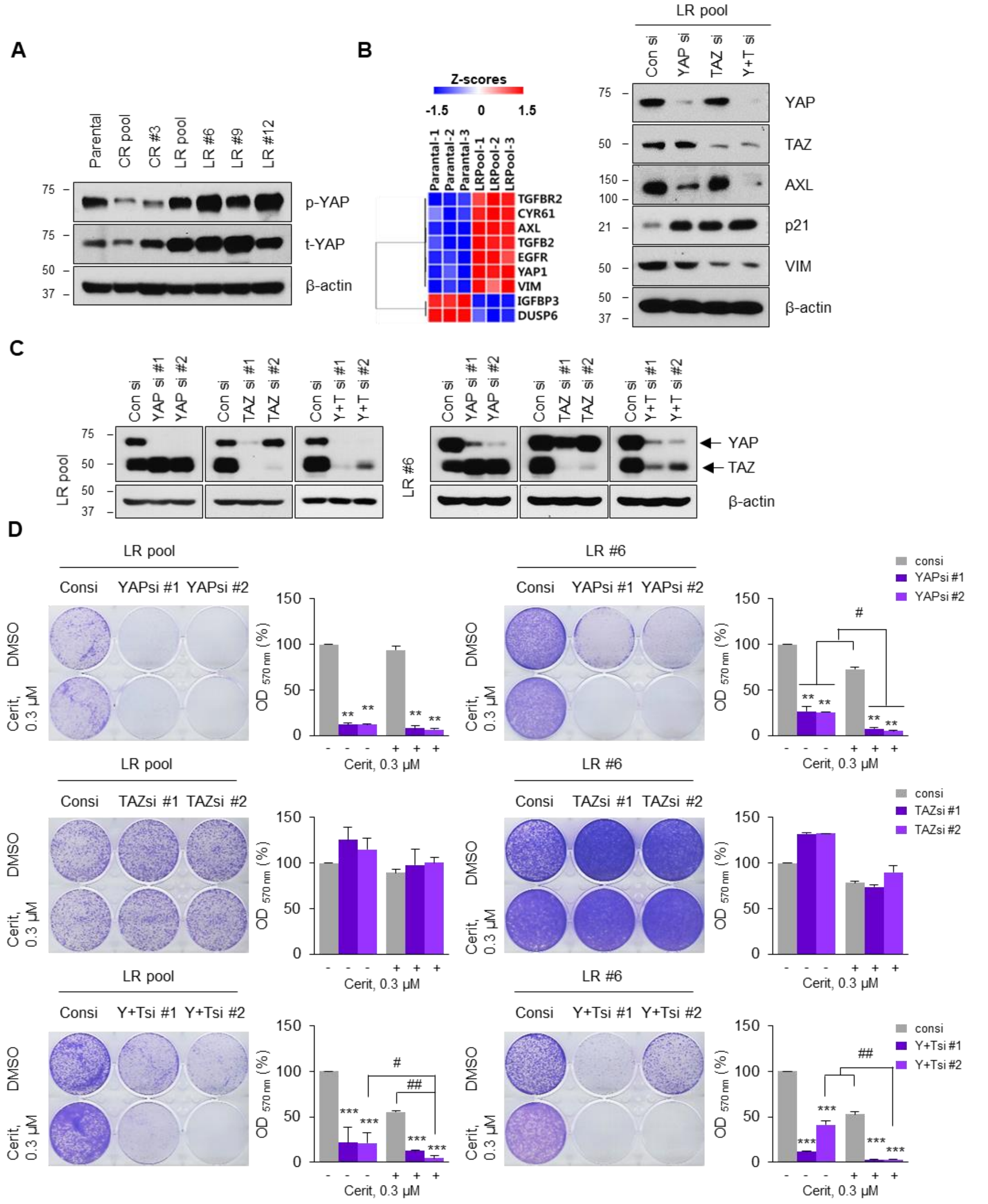

Appendix Figure S9. Effect of YAP/TAZ knockdown on clonogenicity of ceritinib resistant cells.

A Representative immunoblots for the indicated proteins in basal lysates of CR cells (CR pool and CR #3) and LR cells (LR pool, LR #6, LR #9 and LR #12) compared to parental cells.

B Left, Heat-map showing YAP signature from previously published the RNA-seq data in LR pool cells ($P < 0.05$ and fold change>1.5-fold). Right, Representative immunoblots for the indicated proteins in lysates of LR pool cells transiently transfected with siRNAs against negative control, YAP and TAZ.
C Representative immunoblots of the indicated proteins in lysates of LR cells transiently transfected with either negative control siRNA (Con si), YAP siRNAs (two sets of siRNAs against YAP; YAP si#1 and YAP si#2), TAZ siRNAs (two sets of siRNAs against TAZ; TAZ si#1 and TAZ si#2), or combination of TAZ siRNAs with YAP si #2 (Y+T si #1 and Y+T si #2).
D Colony formation in the indicated cells treated with either DMSO or ceritinib 24 h after siRNA transfection. Left, Representative images for crystal violet staining. Right, Quantification for crystal violet staining.

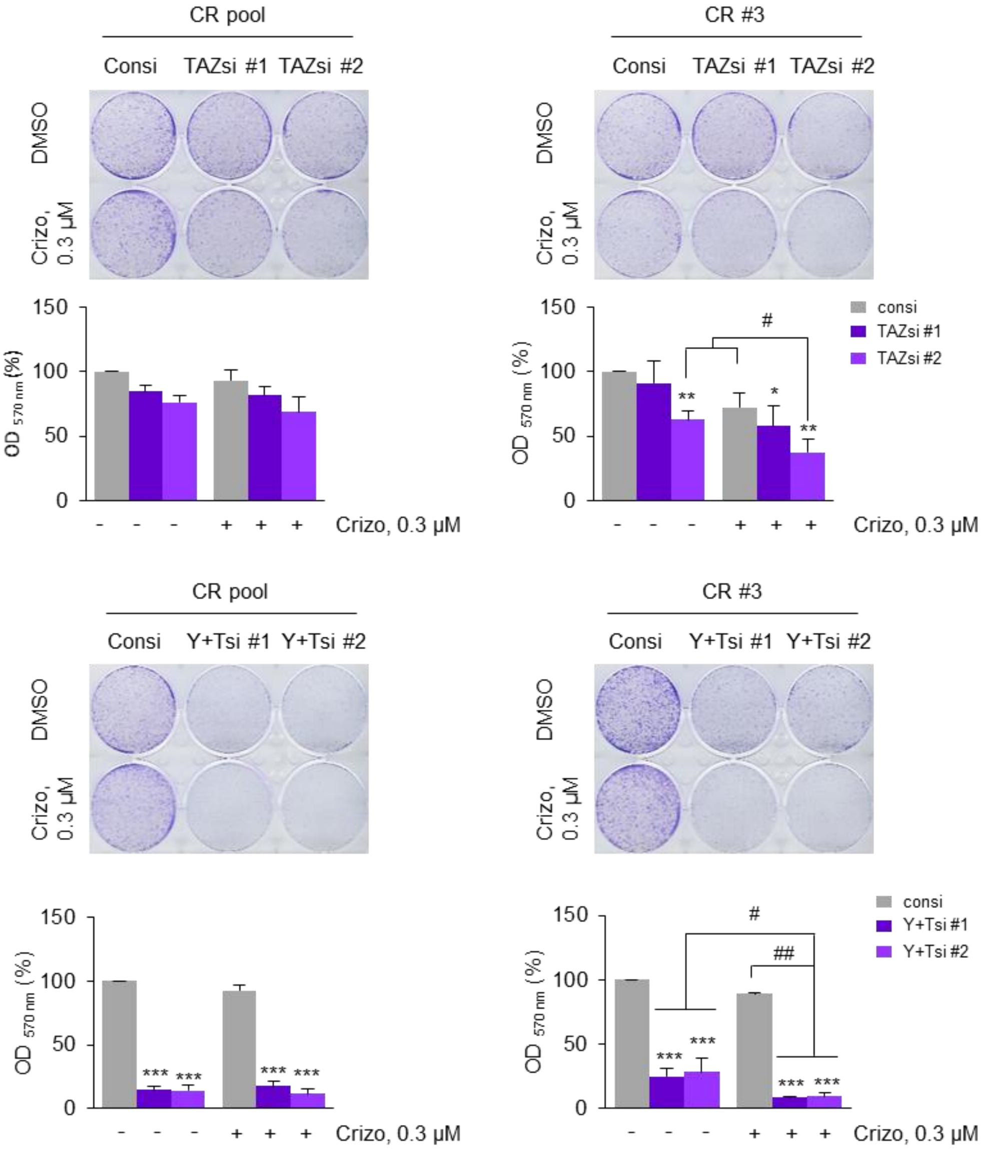

Appendix Figure S10. Effect of YAP/TAZ knockdown on clonogenicity of crizotinib resistant cells.

Colony formation of the indicated cells treated with either DMSO or crizotinib 24 h after siRNA transfection. Upper, Representative images for crystal violet staining. Bottom, Quantification for crystal violet staining.

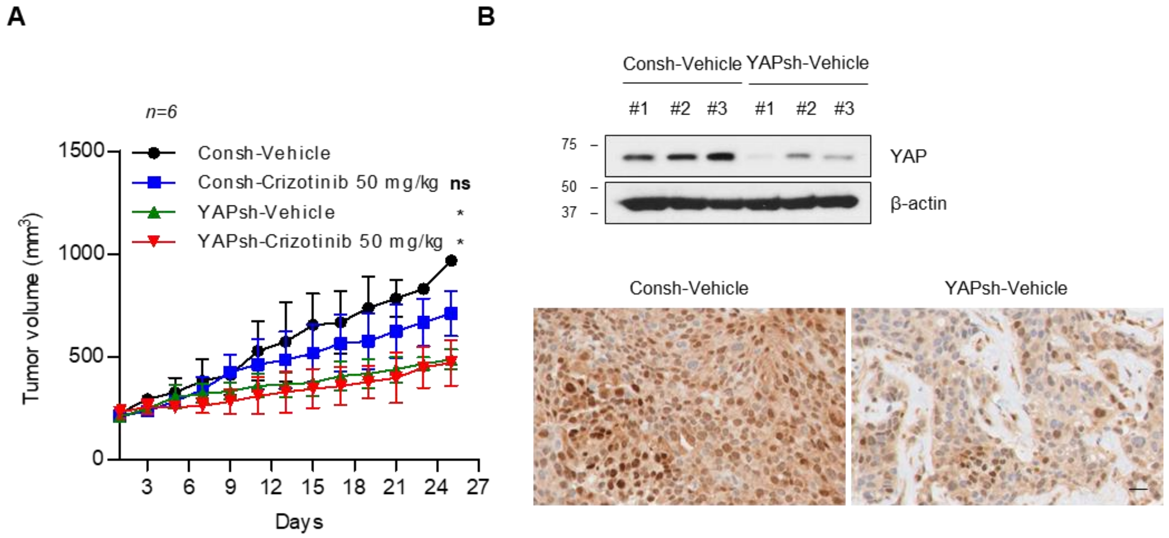

Appendix Figure S11. Effect of YAP knockdown on tumor growth in Con shRNA- or YAP shRNA- xenograft

A Tumor growth curve in Con shRNA- or YAP shRNA- stable CR pool cells-derived xenografts during treatment with crizotinib (50 mg/kg). (Kruskal-Wallis followed by Dunn's post hoc test: *$P < 0.05$ vs. vehicle. ns, not significant. n = 6.)

B Representative immunoblot of YAP protein and representative images of YAP IHC staining in Con shRNA- or YAP shRNA- vehicle tumors at the end of experiment of drug treatment. Scale bar, 20 ㎛

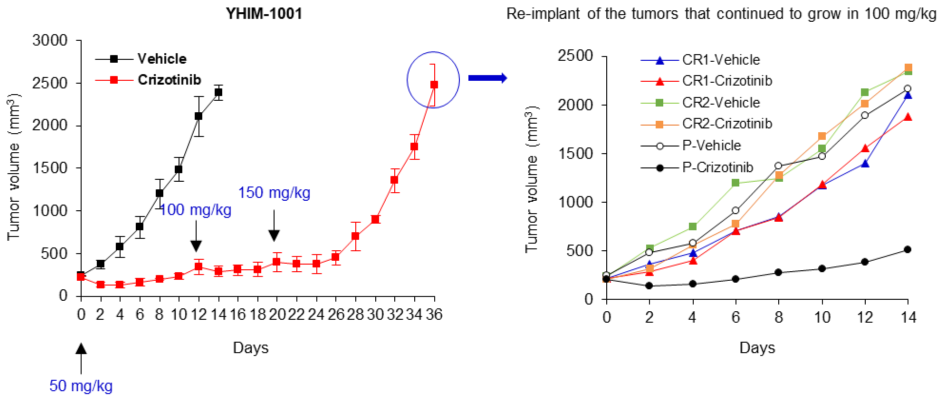

Appendix Figure S12. Establishment of crizotinib-resistant PDX.

Crizotinib-resistant xenograft model (YHIM-1001CR) was derived by continuous *in vivo* crizotinib treatment as described in Methods. Left, Tumor growth curve in time of the first generation of tumors. Right, After one treatment course, Tumor growth curve following vehicle or 150 mg/kg crizotinib treatment in re-implanted tumors.

Appendix Table S1. TP53 mutational status in crizotinib-resistant cells compared with H3122 parental cells

Variant	Alteration	Parantal	CR pool	CR #1	CR #3
A159D	c.476C>A				
A159P	c.475G>C				
A159V	c.476C>T				
**E285V**	**c.854A>T**	**Detected**	**Detected**	**Detected**	**Detected**
G245A	c.734G>C				
G245C	733G>T				
G245D	c.734G>A				
G245F	c.733_734GG>TT				
G245N	c.733_734GG>AA				
G245R	c.733G>C				
G245S	c.733G>A				
G245V	c.734G>T				
R158C	c.472C>T				
R158G	c.472C>G				
R158H	c.473G>A				
R158L	c.473G>T				
R158P	c.473G>C				
R158S	c.472C>A				
R175C	c.523C>T				
R175G	c.523C>G				
R175H	c.524G>A				
R175L	c.524G>T				
R248G	c.742C>G				
R248L	c.743G>T				
R248P	c.743G>C				
R248Q	c.743G>A				
R248W	c.742C>T				
R249M	c.746G>T				

R249S	c.747G>T				
R273C	c.817C>T				
R273G	c.817C>G				
R273H	c.818G>A				
R273L	c.818G>T				
R273P	c.818G>C				
R273S	c.817C>A				
R280K	c.839G>A				
R282W	c.844C>T				
V157D	c.470T>A				
V157F	c.469G>T				
V157G	c.470T>G				
V157L	c.469G>C				
Y220C	c.659A>G				

Appendix Table S2. Variants identified in available post-ALK TKI biopsy samples

Patients	Gene	cDNA change	AA Change
3	RB1	c.959G>A	p.R320Q
	TP53	c.98C>G	p.P33R
	KDR	c.1416A>T	p.Q472H
4	MET	c.1124A>G	p.N375S
	TP53	c.98C>G	p.P33R
	KDR	c.1416A>T	p.Q472H
5	MET	c.1124A>G	p.N375S
	TP53	c.740A>G	p.E247G
	TP53	c.98C>G	p.P33R
	KDR	c.1416A>T	p.Q472H
7	TP53	c.98C>G	p.P33R
	KDR	c.1416A>T	p.Q472H
	PTEN	c.511C>G	p.L171V
8	TP53	c.521G>A	p.R174Q
	KDR	c.1416A>T	p.Q472H
9	ALK	c.3586C>A	p.L1196M
	TP53	c.464T>G	p.L155R
	KDR	c.1416A>T	p.Q472H
10	PTEN	c.A722G	p.Y241C

Appendix Table S3. Primer sequences Sanger Sequencing, site-directed mutagenesis

**Primer sequences**	
**EML4-ALK (For Sanger Sequencing)**	
EML4 (Forward)	5’-TTAGCATTCTTGGGGAATGG-3’
ALK (Reverse)	5’-GCCTGTTGAGAGACCAGGAC-3’
**YAP1_S127A (For site-directed mutagenesis from pBABE-YAP1)**	
Forward	5’-GCATGTTCGAGCTCATGCCTCTCCAGCTTCTC-3’
Reverse	5’-GAGAAGCTGGAGAGGCATGAGCTCGAACATGC-3’
**For exchange the backbone vector from retrovirus-based to lentivirus-based**	
YAP-EcoR Ⅰ (Forward)	5’-TTCGAATTCATGGACCCCGGGCAGCAGCCG-3’
YAP-Xba Ⅰ (Reverse)	5’-TTATCTAGACTATAACCATGTAAGAAAGCT-3’

Appendix Table S4. Information of antibodies used for immunoblotting, immunofluorescence and immunohistochemistry.

Lists of antibody				
**Antibody**	**Dilutions**	**Molecular weight**	**Company**	**Catalogs**
phospho-ALK	1:1000	120 kDa	Cell Signaling Technology	#3341
ALK	1:1000	120 kDa	Cell Signaling Technology	#3633S
phospho-AKT	1:1000	60 kDa	Cell Signaling Technology	#9271
AKT	1:1000	60 kDa	Cell Signaling Technology	#9272
phospho-ERK	1:1000	42, 44 kDa	Cell Signaling Technology	#9101
ERK	1:1000	42, 44 kDa	Cell Signaling Technology	#9106
cleaved PARP	1:1000	89 kDa	Cell Signaling Technology	#9541
cleaved Caspase3	1:1000	17, 19 kDa	Cell Signaling Technology	#9661
p21	1:1000	21 kDa	Cell Signaling Technology	#2947
phospho-YAP	1:1000	65-75 kDa	Cell Signaling Technology	#4911
YAP	1:1000	65-75 kDa	Cell Signaling Technology	#4912
TAZ	1:1000	50 kDa	Cell Signaling Technology	#4883
YAP/TAZ	1:1000	50,70 kDa	Cell Signaling Technology	#8418
LaminA/C	1:1000	63,74 kDa	Cell Signaling Technology	#4777
LATS	1:1000	140 kDa	Cell Signaling Technology	#9153
CYR61	1:1000	41 kDa	Cell Signaling Technology	#14479

Axl	1:1000	138 kDa	Cell Signaling Technology	#8661
Vimentin	1:1000	50, 57 kDa	Cell Signaling Technology	#3932
CyclinD1	1:1000	37 kDa	Santa Cruz Biotechnology	sc-8396
EGFR	1:1000	170 kDa	Santa Cruz Biotechnology	sc-373746
TGFβR2	1:1000	64/67 kDa	Santa Cruz Biotechnology	sc-400
IGFBP3	1:1000	40/44 kDa	Santa Cruz Biotechnology	sc-9028
Tubulin	1:1000	50-55 kDa	Abcam	AB18207
DUSP6	1:1000	42 kDa	Abcam	AB76310
Anti-β-actin antibody	1:10000	42 kDa	Sigma Chemical Co	A3854
YAP (IF, IHC)	1:100	-	Santa Cruz Biotechnology	sc101199
donkey anti-rabbit IgG-HRP	1:2000	-	Santa Cruz Biotechnology	sc-2077
donkey anti-mouse IgG-HRP	1:2000	-	Santa Cruz Biotechnology	sc-2096

Appendix Table S5. Summary of statistical test and exact *P*-values for each specific figure (If *P*-value was <0.0001 the exact value is not given by the analysis software)

Figure	Compared pairs	*P*-value	Test used	significance
1D	Veh vs Crizo in parental	*P*=0.0016	Kruskal-Wallis with Dunn	**
	Veh vs Ceriva in parental	*P*=0.9891		ns
	Veh vs Crizo+Ceriva in parental	*P*=0.0022		**
	Veh vs Crizo in CR pool	*P*=0.0518		ns
	Veh vs Ceriva in CR pool	*P*<0.0001		**
	Veh vs Crizo+Ceriva in CR pool	*P*<0.0001		**
	Crizo vs Crizo+Ceriva in CR pool	*P*=0.0081		##
	Ceriva vs Crizo+Ceriva in CR pool	*P*=0.9999		ns
1E	Con vs Ceriva in parental	*P*=0.0415	ANOVA with Tukey	*
	Con vs Ceriva in CR pool	*P*=0.0027		**
	Ceriva vs Ceriva+MVA in CR pool	*P*=0.0045		##
	Ceriva vs Ceriva+GGPP in CR pool	*P*=0.0052		##
	Con vs Ceriva in CR #1	*P*=0.0066		**
	Ceriva vs Ceriva+MVA in CR #1	*P*=0.0457		#
	Ceriva vs Ceriva+GGPP in CR #1	*P*=0.0451		#
	Con vs Ceriva in CR #3	*P*<0.0001		**
	Ceriva vs Ceriva+MVA in CR #3	*P*<0.0001		##

	Ceriva vs Ceriva+GGPP in CR #3	*P*=0.0014		##
3A	0 day vs. 21 days in pLVX	*P*=0.3845	Kruskal-Wallis with Dunn	ns
	0 day vs. 21 days in YAP-WT	*P*=0.0002		***
	0 day vs. 21 days in YAP-S127A	*P*<0.0001		***
	21 days in pLVX vs 21 days in YAP-WT	*P*=0.0187		#
	21 days in pLVX vs 21 days in YAP-S127A	*P*<0.0001		###
	21 days in YAP-WT vs 21 days in YAP-S127A	*P*=0.0082		§§
3B	Veh vs Crizo in pLVX	*P*=0.0027	Kruskal-Wallis with Dunn	**
	Veh vs Ceriva in pLVX	*P*=0.3748		ns
	Veh vs Crizo in YAP-WT	*P*=0.5128		ns
	Veh vs Ceriva in YAP-WT	*P*=0.0001		**
	Veh vs Crizo+Ceriva in YAP-WT	*P*<0.0001		**
	Crizo vs Crizo+Ceriva in YAP-WT	*P*=0.0002		##
	Ceriva vs Crizo+Ceriva in YAP-WT	*P*=0.0258		#
	Veh vs Crizo in YAP-S127A	*P*=0.2329		ns
	Veh vs Ceriva in YAP-S127A	*P*=0.0022		**
	Veh vs Crizo+Ceriva in YAP-S127A	*P*=0.0012		**
	Crizo vs Crizo+Ceriva in YAP-S127A	*P*=0.1088		ns
	Ceriva vs Crizo+Ceriva in YAP-S127A	*P*=0.9931		ns

4A	Consi-DMSO vs YAPsi #1-DMSO in CR pool	*P*=0.0014	ANOVA with Tukey	**
	Consi-DMSO vs YAPsi #2-DMSO in CR pool	*P*=0.0023		**
	Consi-DMSO vs YAPsi #1-Crizo in CR pool	*P*=0.0001		**
	Consi-DMSO vs YAPsi #2-Crizo in CR pool	*P*=0.0001		**
	YAPsi #2-DMSO vs YAPsi #2-Crizo in CR pool	*P*=0.0127		#
	Consi-Crizo vs YAPsi #2-Crizo in CR pool	*P*=0.0012		##
	Consi-DMSO vs YAPsi #1-DMSO in CR #3	*P*=0.0014		**
	Consi-DMSO vs YAPsi #2-DMSO in CR #3	*P*=0.0020		**
	Consi-DMSO vs YAPsi #1-Crizo in CR #3	*P*=0.0001		**
	Consi-DMSO vs YAPsi #2-Crizo in CR #3	*P*=0.0001		**
	YAPsi #2-DMSO vs YAPsi #2-Crizo in CR #3	*P*=0.0428		#
	Consi-Crizo vs YAPsi #2-Crizo in CR #3	*P*=0.0013		##
4C	Consh vs YAPsh in left	*P*=0.0029	Kruskal-Wallis with Dunn	**
	0 day vs 21 day in Consh	*P*<0.0001		**
	0 day vs 21 day in YAPsh	*P*=0.3200		ns
	21 day in Consh vs 21 day in YAPsh	*P*=0.0055		##
4D	Veh vs Crizo	*P*=0.9950	Kruskal-Wallis with Dunn	ns
	Veh vs VP	*P*=0.0037		**
	Veh vs Crizo+VP	*P*=0.0008		**

	Crizo vs Crizo+VP	*P*=0.0021		##
	VP vs Crizo+VP	*P*=0.7221		ns
5A	Veh vs Crizo	*P*=0.9928	Kruskal-Wallis with Dunn	ns
	Veh vs Ceriva	*P*=0.0487		*
	Veh vs VP	*P*=0.0713		ns
	Veh vs Crizo+Ceriva	*P*=0.0228		*
	Veh vs Crizo+VP	*P*=0.0412		*
	Crizo vs Crizo+Ceriva	*P*=0.0341		#
	Ceriva vs Crizo+Ceriva	*P*=0.8812		ns
	Crizo vs Crizo+VP	*P*=0.0412		#
	VP vs Crizo+VP	*P*=0.9841		ns
6B	Pre-TKI vs Post-TKI in left	*P*=0.066	Mann-Whitney	ns
	Pre-TKI vs Post-TKI in right	*P*=0.0015		**
