# Supplementary material for: Targeting YAP to overcome acquired resistance to ALK inhibitors in ALK‐rearranged lung cancer
Source: EMBO Mol Med. 2019 Oct 21;11(12):e10581. doi: 10.15252/emmm.201910581 (PMC6895608; doi:10.15252/emmm.201910581)
Supplement: Supplementary file 2 — Expanded View Figures PDF [file EMMM-11-e10581-s002.pdf]

Expanded View Figures

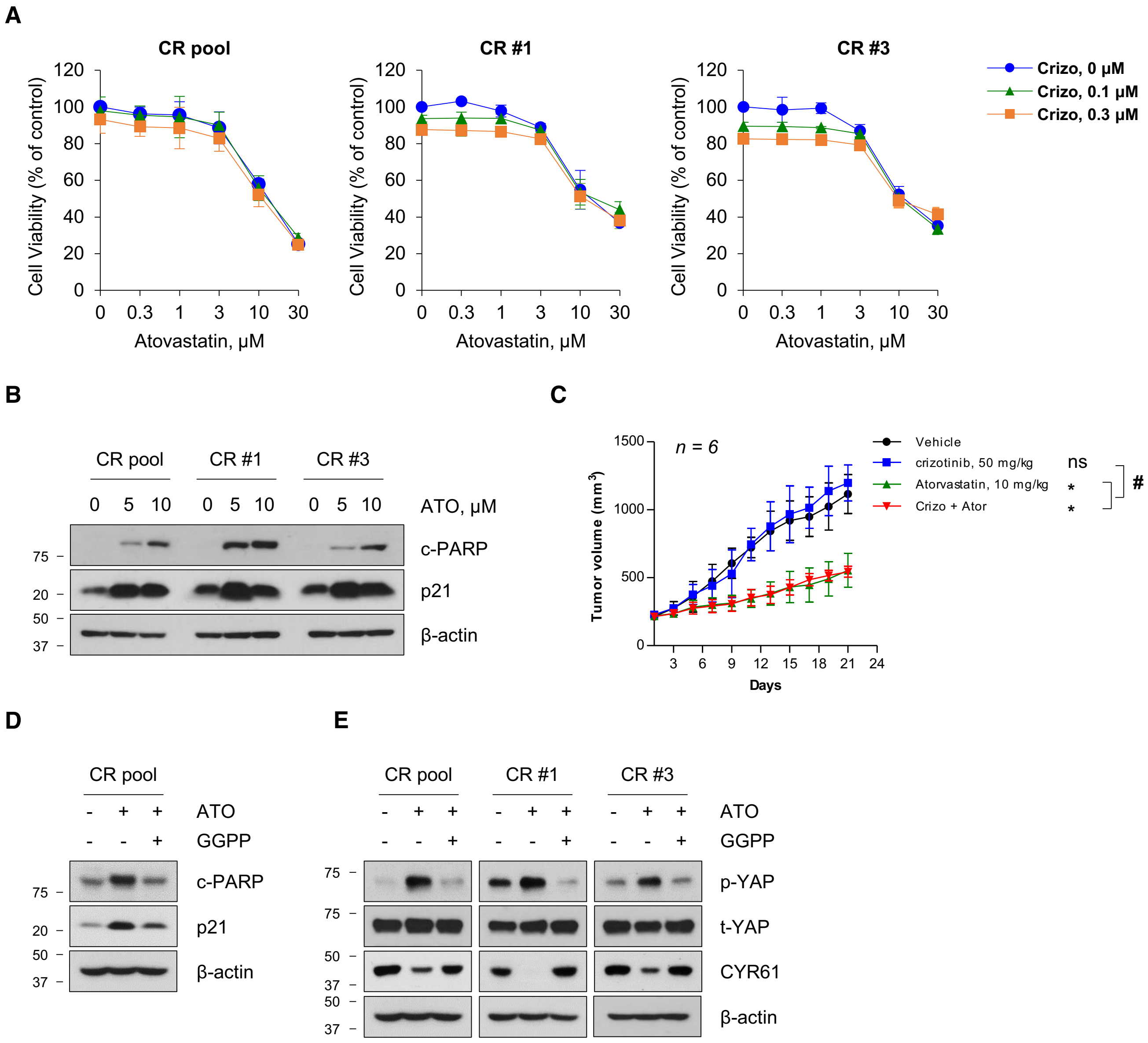

Figure EV1. ***In vitro* and *in vivo* anti-cancer activity of atorvastatin.**

A Cell viability curve in response to combined treatment of simvastatin and crizotinib in parental and CR cells using MTT assays. Data represent means ± SD ($n = 3$).

B Representative immunoblots of the indicated proteins in lysates of cells treated with atorvastatin (ATO) for 24 h.

C Tumor growth curves of CR pool xenografts ($n = 6$) treated with the indicated drugs. Data represent means ± SD (*$P < 0.05$ vs. vehicle, $^{\#}P < 0.05$ vs. crizotinib treatment. ns, not significant; Kruskal–Wallis followed by Dunn's *post hoc* test).

D, E Representative immunoblots of the indicated proteins in cells treated with ATO (5 μM) alone or with GGPP (10 μM) for 24 h.

Data information: Blots are representative of three independent experiments.

Source data are available online for this figure.

 © 2019 The Authors

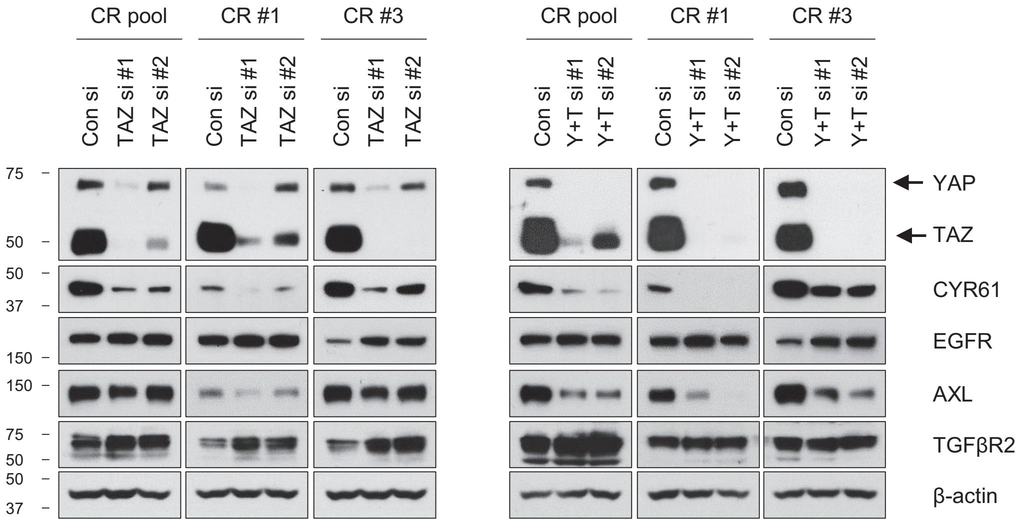

Figure EV2. Effect of TAZ knockdown on expression of yes-associated protein (YAP)-associated genes.

Representative immunoblots of the indicated proteins in lysates of CR cells transiently transfected with either negative control siRNA (Con si), TAZ siRNAs (two sets of siRNAs against TAZ; TAZ si#1 and TAZ si#2), or a combination of TAZ siRNAs with YAP si#2 (Y + T si#1 and Y + T si#2). Blots are representative of three independent experiments.

Source data are available online for this figure.

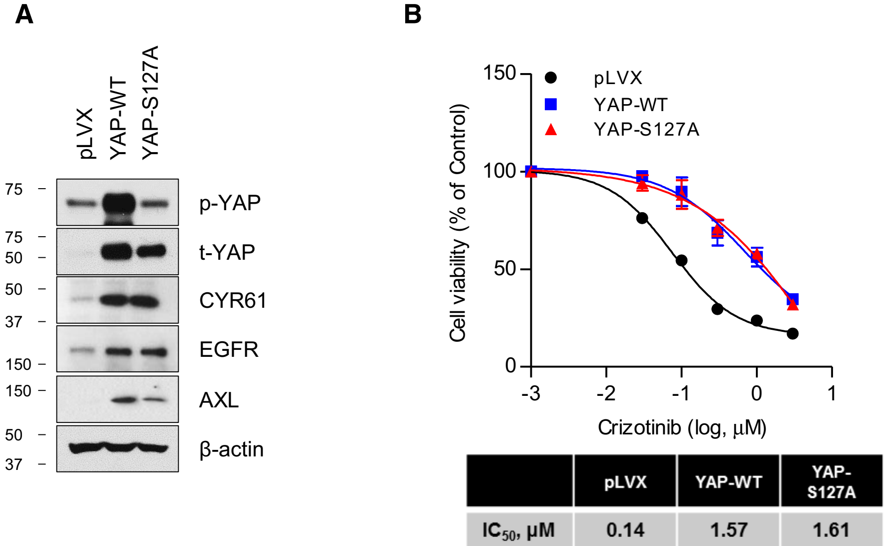

	pLVX	YAP-WT	YAP-S127A
IC_{50}, μM	0.14	1.57	1.61

Figure EV3. Establishment of stable cells overexpressing yes-associated protein (YAP).

A Representative immunoblots of the indicated proteins in cell lysates from stably selected H3122 parental cells after infection with either empty vector (pLVX), YAP wild-type (YAP-WT), or YAP-S127A mutant (YAP-S127A).

B Cell viability curve in response to increasing doses of crizotinib by MTT assay in YAP-overexpressing stable cells. The bottom table shows relative IC_{50} values of each cell line estimated from the cell viability curve. Data represent means ± SD (n = 3).

Source data are available online for this figure.

© 2019 The Authors

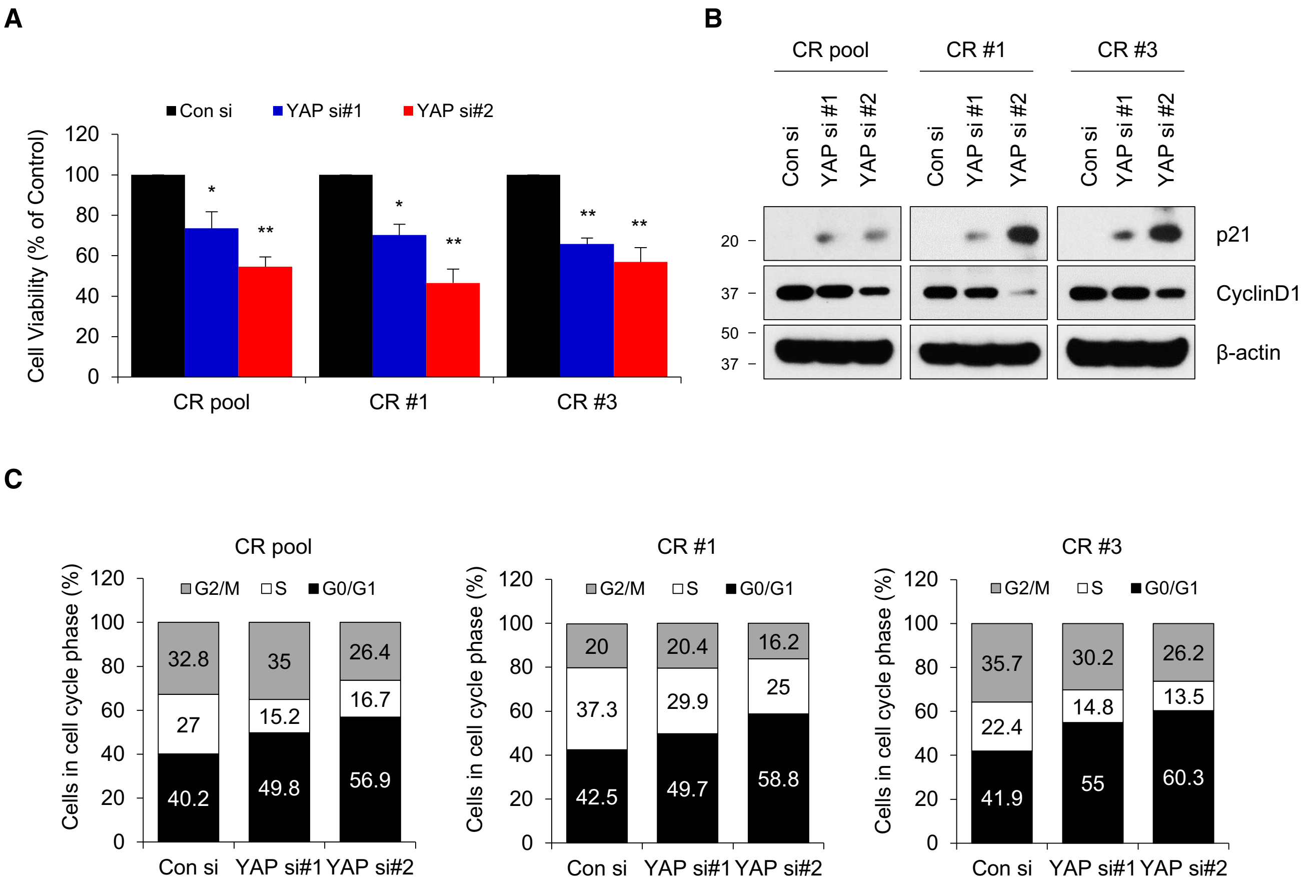

Figure EV4. Effect of yes-associated protein (YAP) knockdown on cell proliferation and cell cycle.

CR cells were transfected with either negative control siRNA (Con si) or YAP siRNAs (two sets of siRNAs against YAP; YAP si#1 and YAP si#2).

A Bar chart showing cell viability of CR cells after transfection using MTT assays. Data represent means ± SD (two-sided Student's *t*-test: *$P < 0.05$, **$P < 0.01$ vs. Con si in each cell line; $n = 4$).

B Representative immunoblots of the indicated proteins 24 h after transfection.

C Cell cycle distribution of cells 24 h after transfection. The percentage of cells (1×10^4) in G0/G1, S, and G2/M phases was quantified.

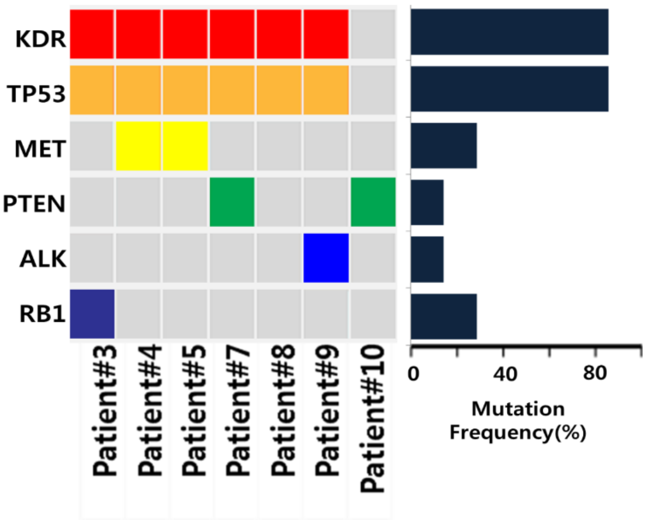

Figure EV5. Variants identified in available post-ALK tyrosine kinase inhibitor (TKI) biopsy samples.

Heat-map showing the mutation patterns of available post-ALK-TKI biopsy samples. Gene mutation frequencies in the samples are shown on the left.

© 2019 The Authors
