# Supplementary figures and images for: Targeting YAP to overcome acquired resistance to ALK inhibitors in ALK‐rearranged lung cancer
Source: EMBO Mol Med. 2019 Oct 21;11(12):e10581. doi: 10.15252/emmm.201910581 (PMC6895608; doi:10.15252/emmm.201910581)

Source data for Figure EV1B, D and E

B

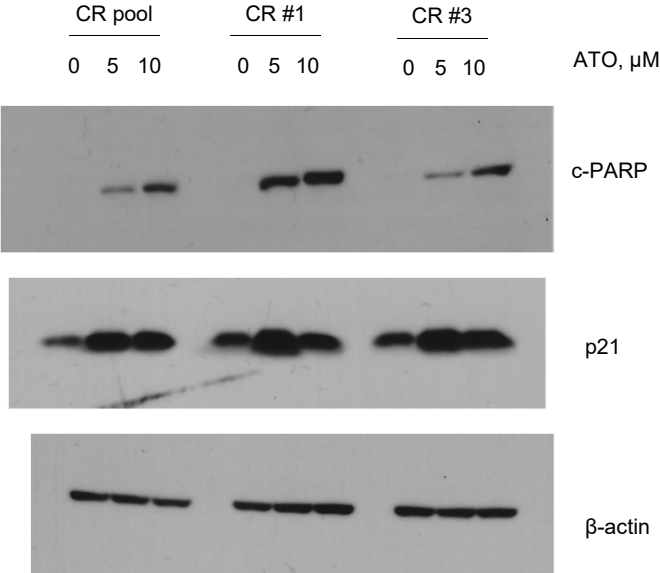

D

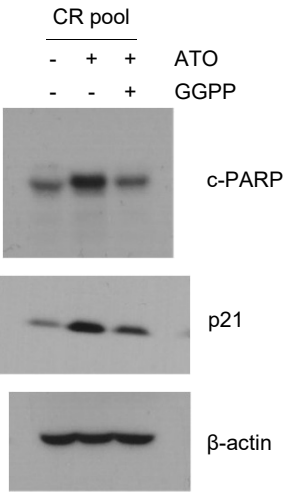

E

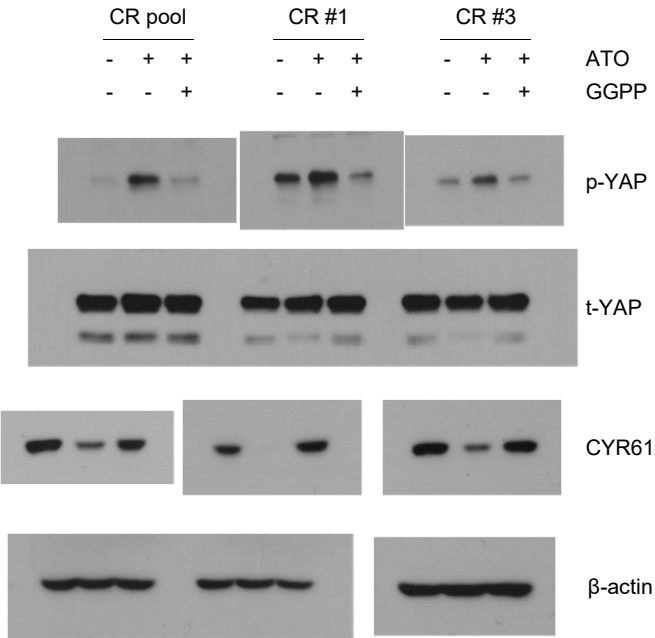

Supplement: Supplementary file 5 — Source Data for Expanded View [file EMMM-11-e10581-s007.zip › EV_source_data/Source_data_for_EV1.pdf]

### Source data for Figure 2D-F

D

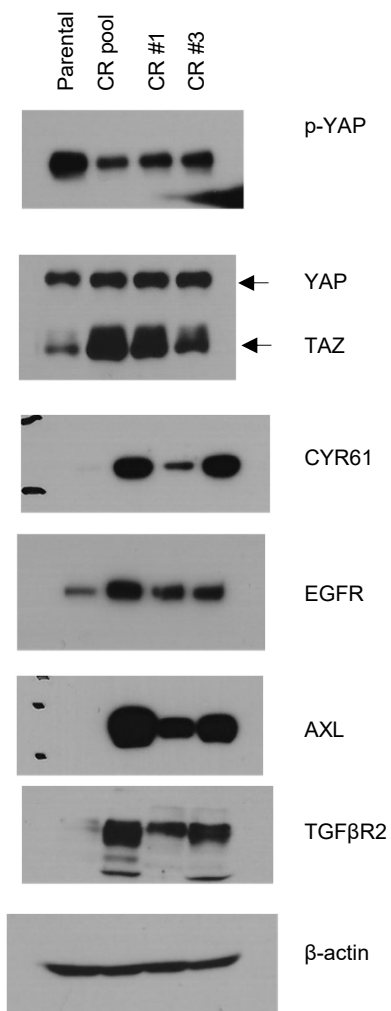

## E

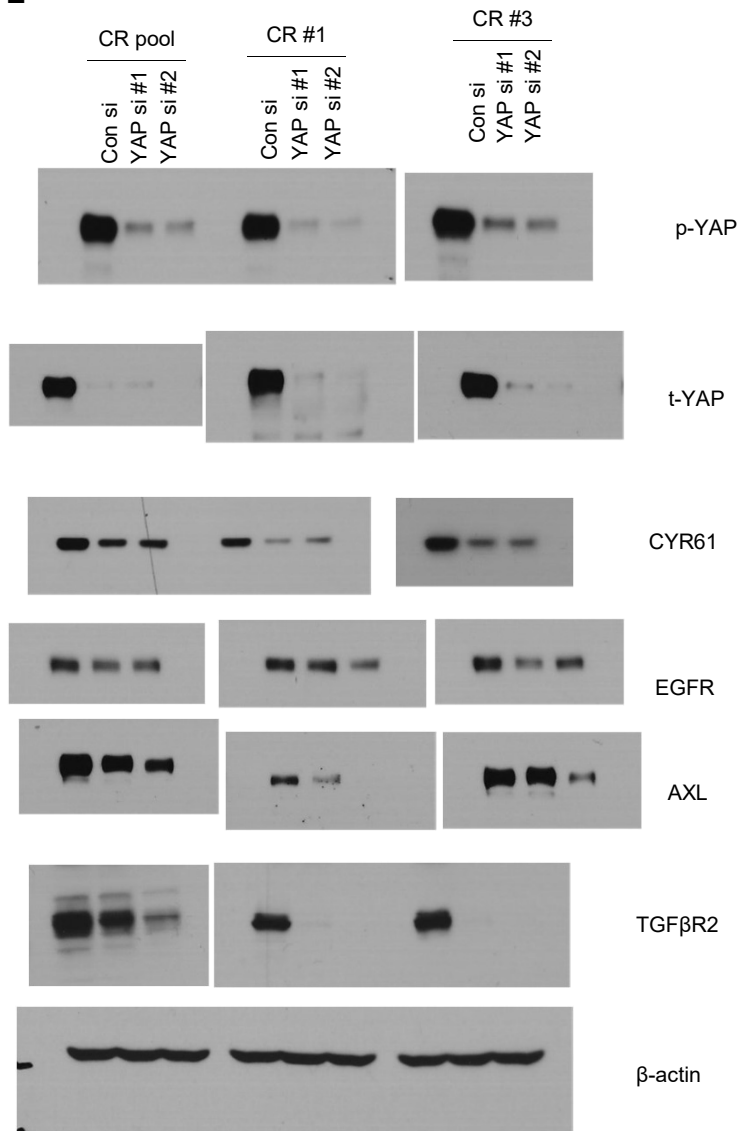

**F**

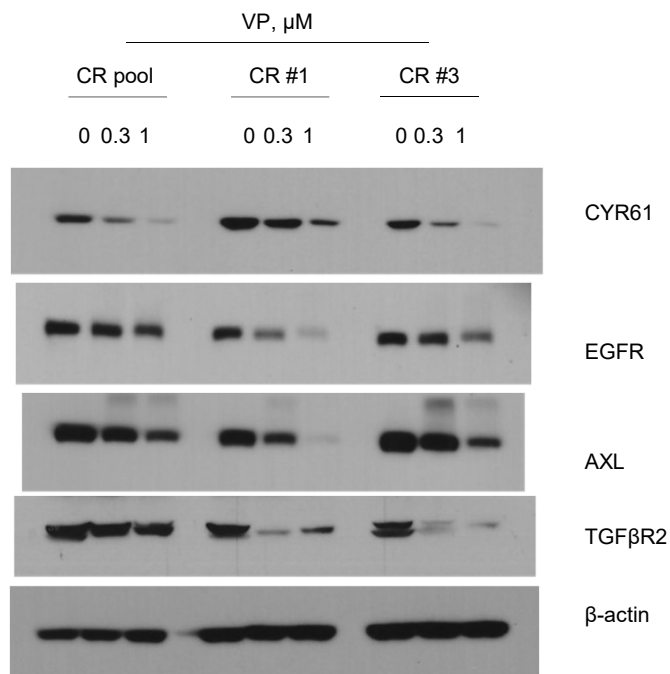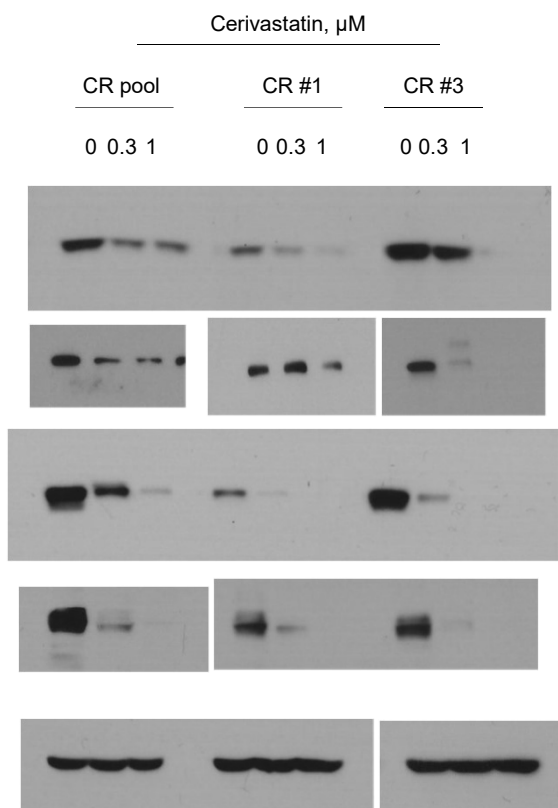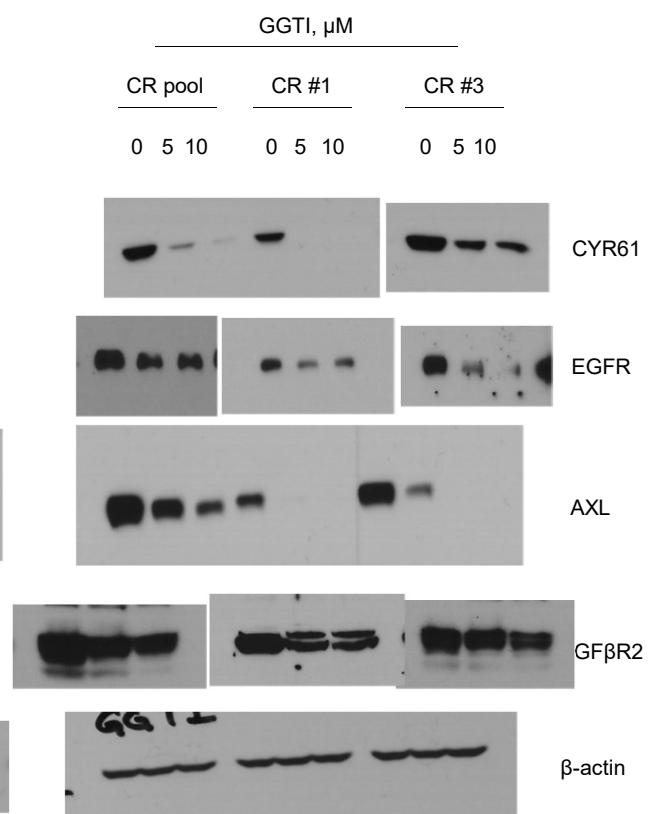

Supplement: Supplementary file 5 — Source Data for Expanded View [file EMMM-11-e10581-s007.zip › EV_source_data/Source_data_for_EV2.pdf]

Source data for Figure EV3A

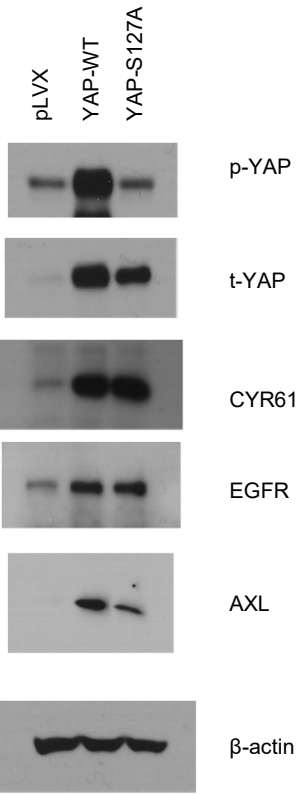

Supplement: Supplementary file 5 — Source Data for Expanded View [file EMMM-11-e10581-s007.zip › EV_source_data/Source_data_for_EV3.pdf]

Source data for Figure 1F

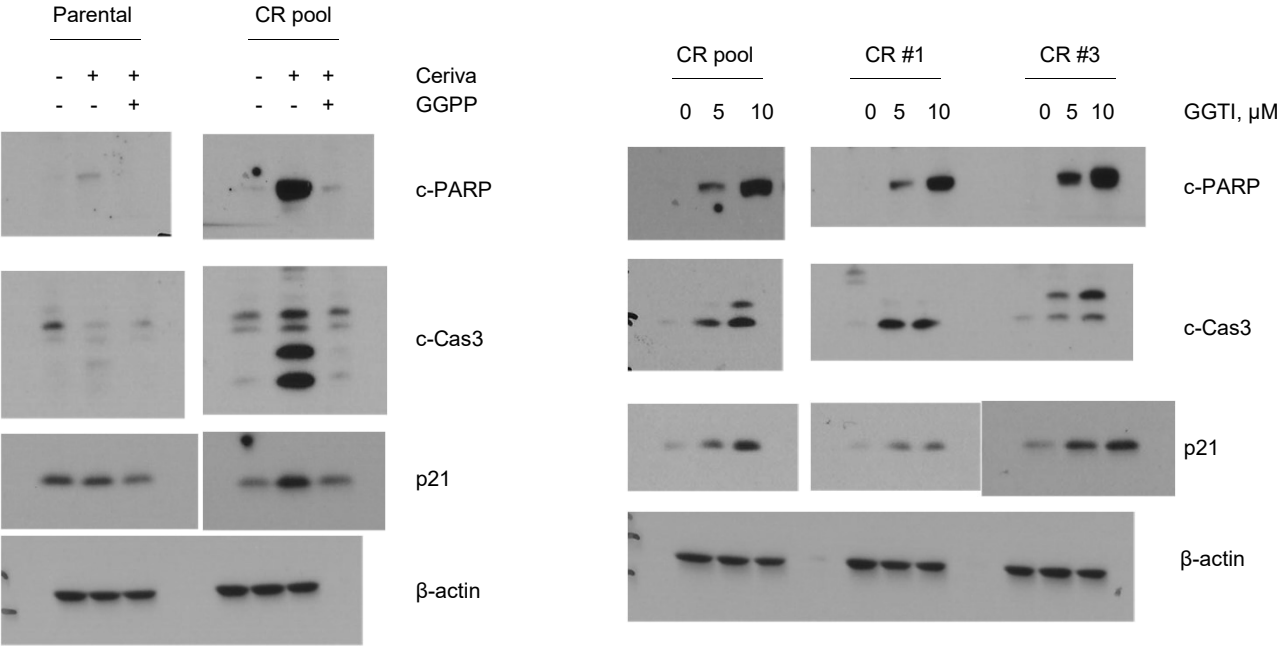

Supplement: Supplementary file 7 — Source Data for Figure 1 [file EMMM-11-e10581-s005.pdf]

### Source data for Figure 2D-F

D

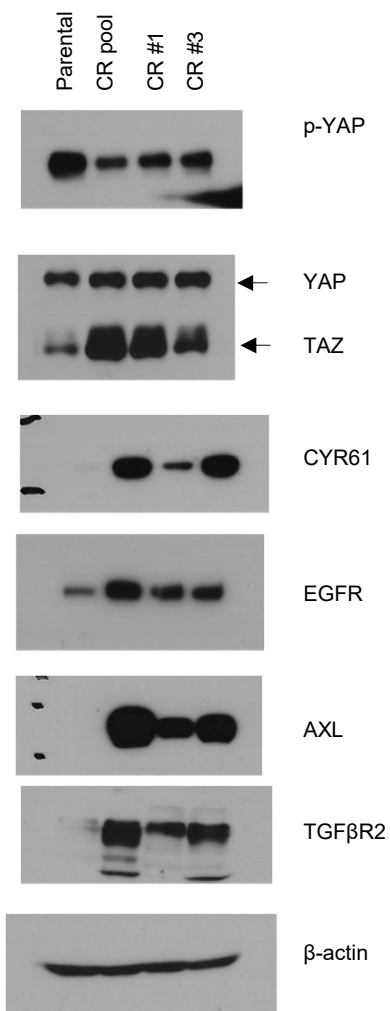

## E

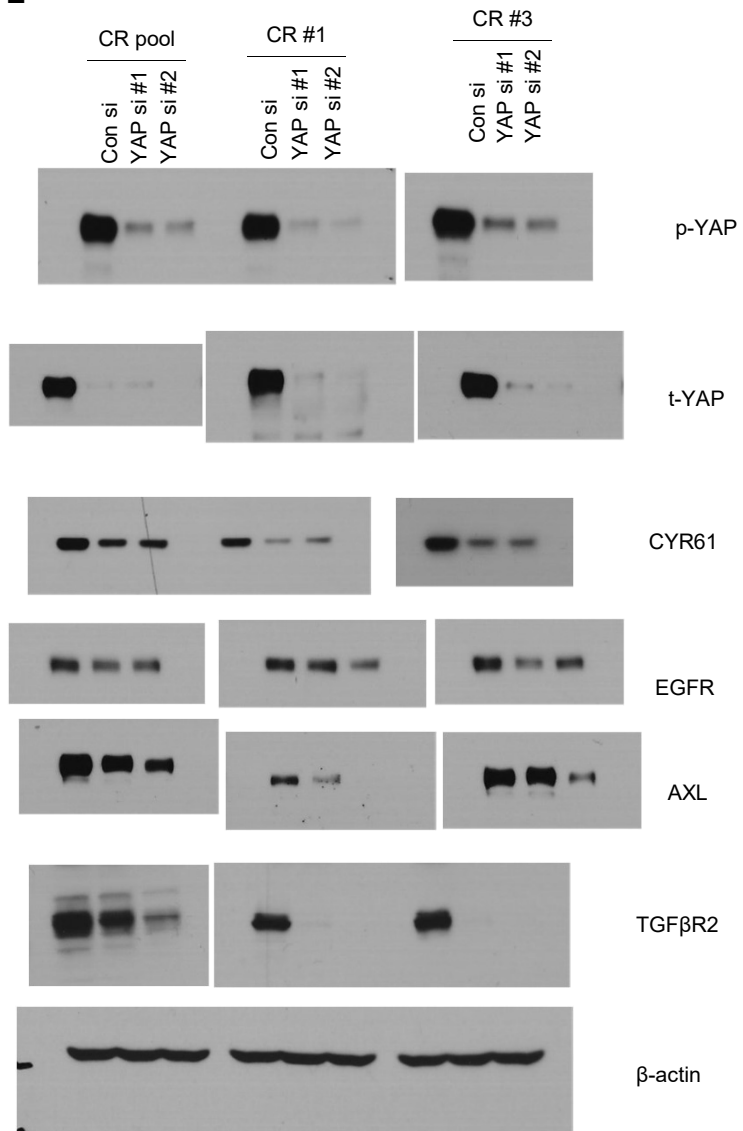

Supplement: Supplementary file 8 — Source Data for Figure 2 [file EMMM-11-e10581-s006.pdf]
